# Supplementary material for: Multi-Omics Analysis Decodes Biosynthesis of Specialized Metabolites Constituting the Therapeutic Terrains of Magnolia obovata
Source: Int J Mol Sci. 2025 Jan 26;26(3):1068. doi: 10.3390/ijms26031068 (PMC11816741; doi:10.3390/ijms26031068)
Supplement: Supplementary file 1 [file ijms-26-01068-s001.zip › FigureS2 Transcriptome expression analysis across seven tissues of Magnolia obovata.pdf]

a

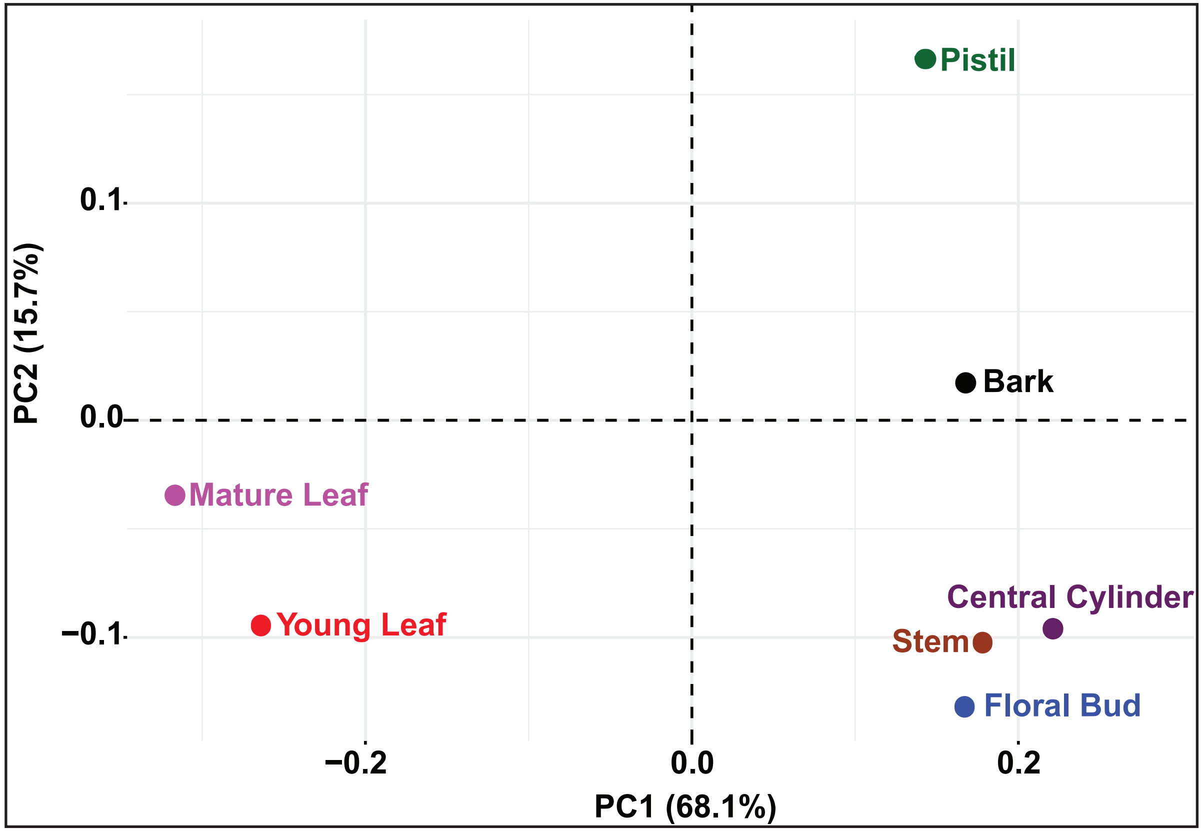

b

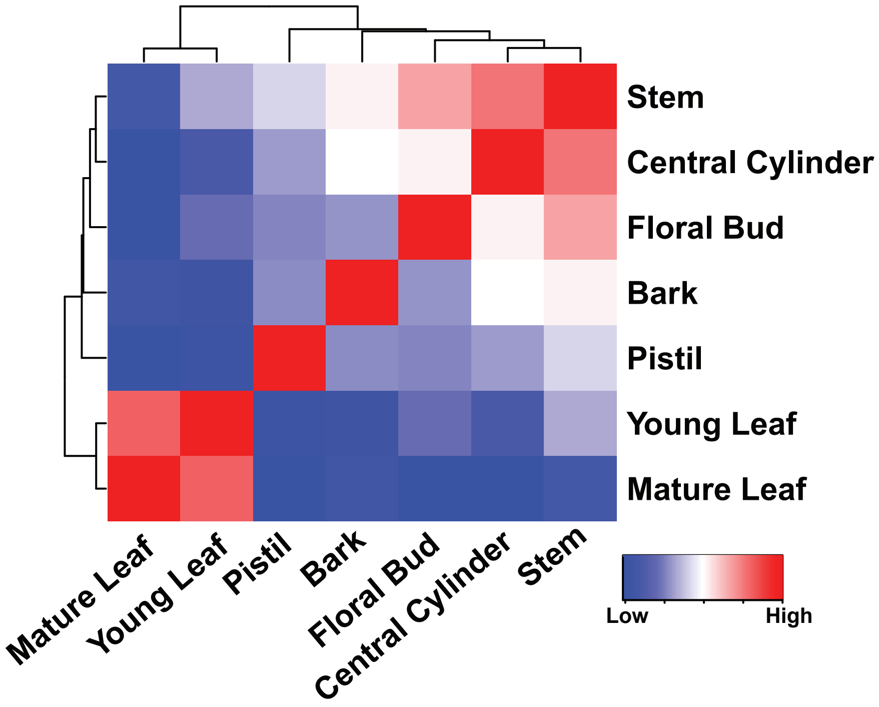

Figure S2: Transcriptome expression analysis across seven tissues of *Magnolia obovata*. **(a)** Unsupervised principal component analysis for seven tissues of *M. obovata*. **(b)** Correlation plot for all seven tissues of *M. obovata*. Transcript abundance analysis was performed and was used to understand the relationship between seven tissues of *M. obovata*. For correlation analysis, Euclidean distance-based hierarchical clustering was performed using expression value of transcripts with non-zero FPKM across seven tissues of *M. obovata*.
