# Supplementary material for: Multi-Omics Analysis Decodes Biosynthesis of Specialized Metabolites Constituting the Therapeutic Terrains of Magnolia obovata
Source: Int J Mol Sci. 2025 Jan 26;26(3):1068. doi: 10.3390/ijms26031068 (PMC11816741; doi:10.3390/ijms26031068)
Supplement: Supplementary file 1 [file ijms-26-01068-s001.zip › FigureS3 Representation of phenylpropanoid biosynthesis pathway by transcripts included in T12 module.pdf]

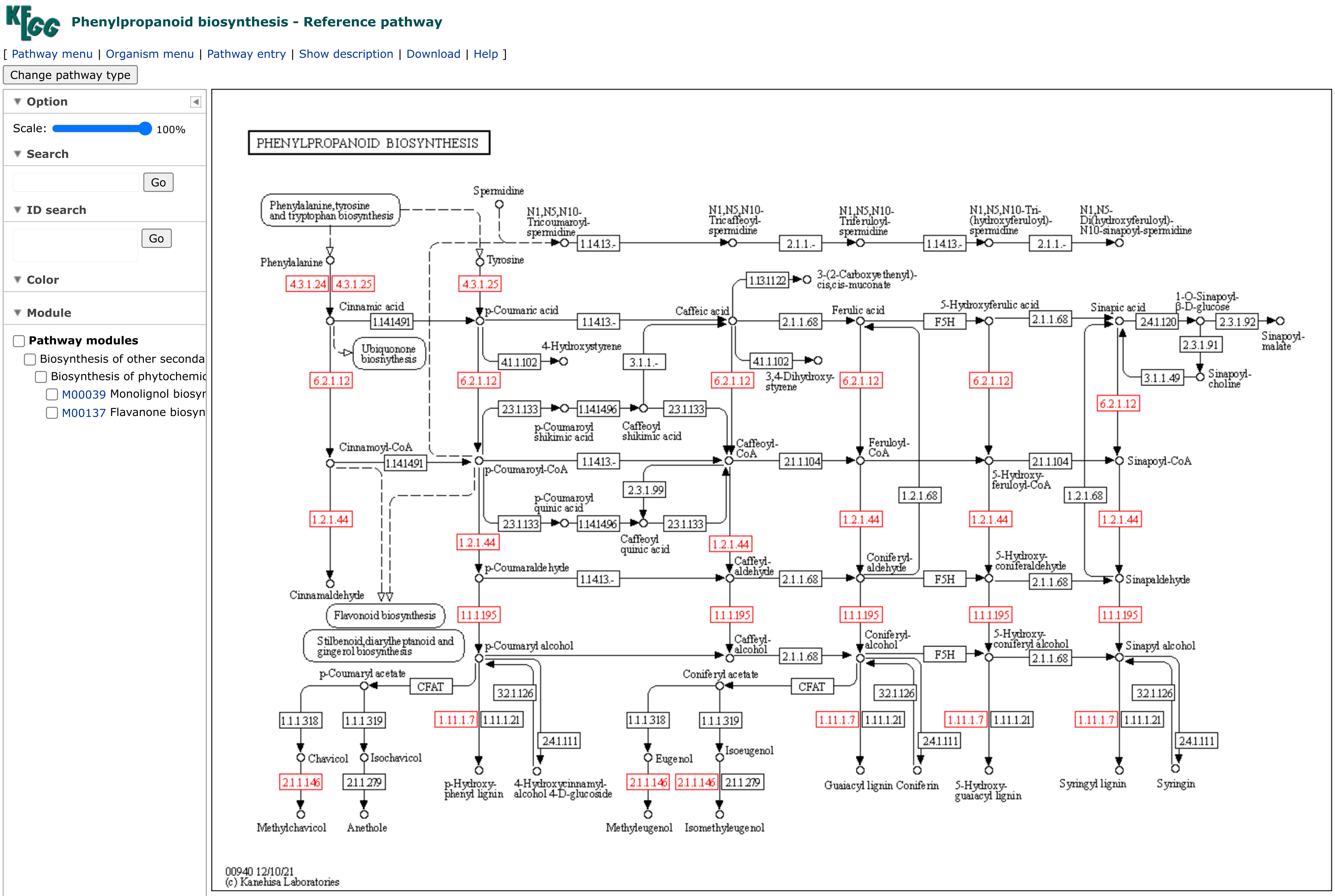

Figure S3: Representation of phenylpropanoid biosynthesis pathway by transcripts included in T12 module sharing high correlation with metabolite module M4 in *Magnolia obovata*. The transcripts grouped in the TransM12 modules were filtered and KEGG pathway mapping was performed using OmicsBox software.
