# Supplementary material for: Multi-Omics Analysis Decodes Biosynthesis of Specialized Metabolites Constituting the Therapeutic Terrains of Magnolia obovata
Source: Int J Mol Sci. 2025 Jan 26;26(3):1068. doi: 10.3390/ijms26031068 (PMC11816741; doi:10.3390/ijms26031068)
Supplement: Supplementary file 1 [file ijms-26-01068-s001.zip › TableS5 BUSCO statistics summary for the de novo transcriptome assembly of Magnolia obovata.docx]

| BUSCO statistics summary | |
| --- | --- |
| Lineage dataset used | viridiplantae_odb10 |
| Number of genomes searched | 57 |
| Number of BUSCOs included in search | 425 |
| Complete Single copy BUSCOs identified | 141 (33.18%) |
| Complete Duplicated BUSCOs identified | 279 (65.65%) |
| Fragmented BUSCOs identified | 5 (1.18%) |
| Missing BUSCOs | 0 (0%) |

**Supplementary Table 5.** BUSCO statistics summary for the *de novo* transcriptome assembly of *Magnolia obovata.*
