# Supplementary material for: Multi-Omics Analysis Decodes Biosynthesis of Specialized Metabolites Constituting the Therapeutic Terrains of Magnolia obovata
Source: Int J Mol Sci. 2025 Jan 26;26(3):1068. doi: 10.3390/ijms26031068 (PMC11816741; doi:10.3390/ijms26031068)
Supplement: Supplementary file 1 [file ijms-26-01068-s001.zip › FigureS1 Characterization of the de novo transcriptome assembly of Magnolia obovata.pdf]

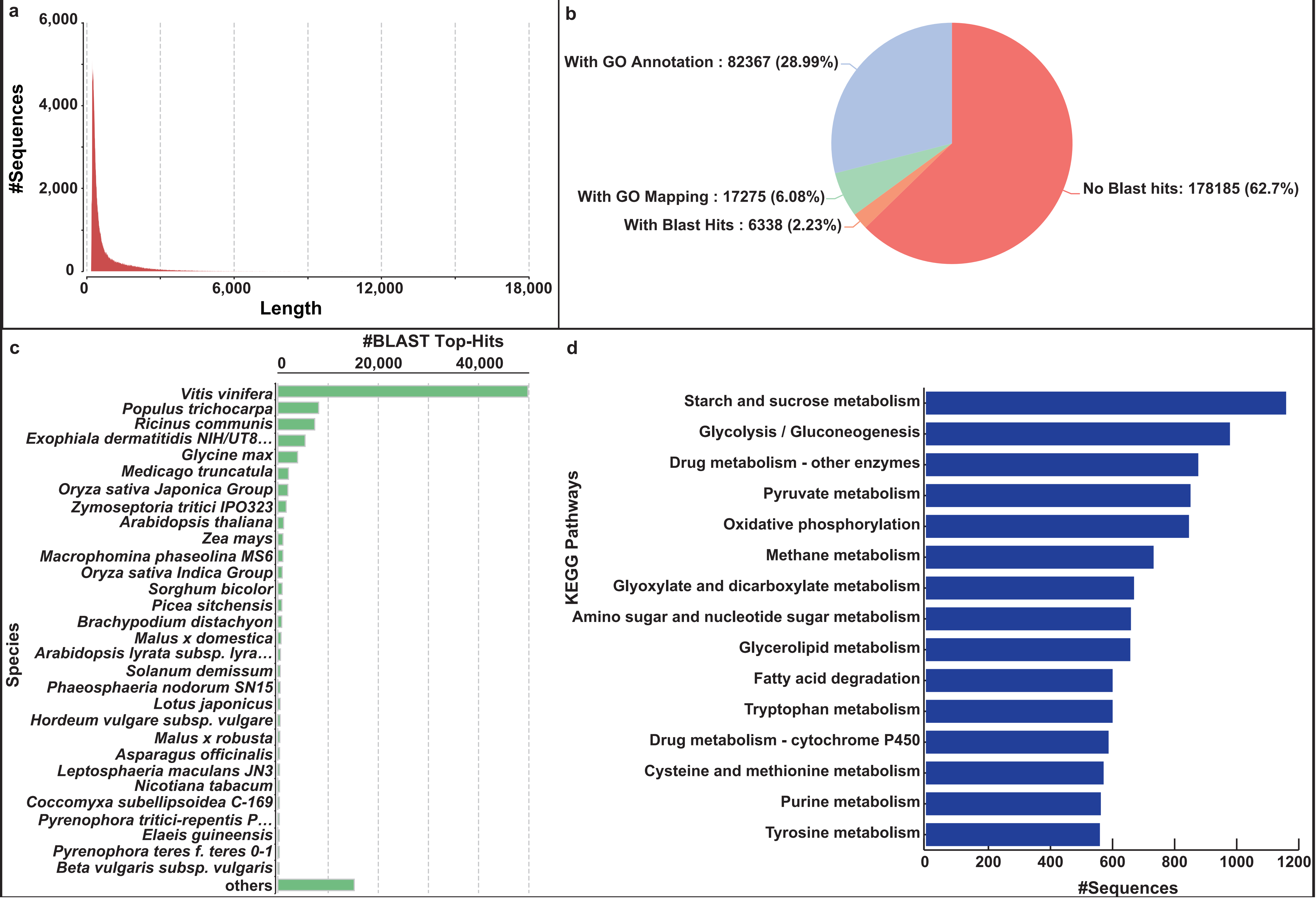

Figure S1: Characterization of the *de novo* transcriptome assembly of *Magnolia obovata*. (a) Length distribution of the assembled transcripts of *M. obovata*. **(b)** Distribution of BLAST search results. **(c)** Top-hit species distribution. **(d)** Top 15 KEGG pathways based on the number of transcripts being mapped.
